# Exploring Exemplary Optoelectronic and Charge Transport Properties of KCuX(X=Se,Te)

**DOI:** 10.1038/s41598-018-31300-0

**Published:** 2018-08-30

**Authors:** Atahar Parveen, G. Vaitheeswaran

**Affiliations:** 1 Advanced Centre of Research in High Energy Materials (ACRHEM), University of Hyderabad, Prof. C. R. Rao Road, Gachibowli, Hyderabad, Telangana 500046 India; 2 School of Physics, University of Hyderabad, Prof. C. R. Rao Road, Gachibowli, Hyderabad, Telangana 500046 India

## Abstract

We report the electronic structure, optical and charge transport properties of the unexplored ternary Zintl phases KCuX(X=Se,Te) from the first principles calculations employing the full-potential linearized augmented plane-wave (FLAPW) method with the Tran Blaha modified Becke-Johnson (TBmBJ) potential. It is demonstrated that the materials are direct band gap (1.13, 1.38 eV) semiconductors with covalent bonding between Cu and (Se/Te). The calculated low effective mass and high carrier mobility (over 10^5^ *cm*^2^/V.s) accentuate that KCuX have good carrier transport and the materials may have possible applications in solar cell absorbers and nanoelectronic devices. Absorption spectra indicates that the ternary crystals are UV-A light absorbers and could be useful in photovoltaic and photodetector applications. A study on the effect of pressure (till 5 GPa) is carried out in order to further explore the materials for their electronic band gaps and charge transport properties as they are proposed to be useful in future contemporary electronic devices. It is observed that pressure enhances the intrinsic carrier mobility and thermal stability of KCuX, indicating that the materials can withstand robust external conditions.

## Introduction

Semiconductors with narrow band gap have been of great interest since last four decades for their fundamental physics and utility in infrared devices, LEDs, infrared lasers and thermophotovoltaics^1^. The ternary copper-based systems with direct band gap are of particular interest for their potential applications as thin-film photovoltaic absorbers, thermoelectric components, hybrid photodetectors, solar cell absorbers, photocatalysts for solar water splitting and strong optical absorption of 10^4^ *cm*^−1^ in the visible region for solar energy conversion^2^. Also, in Cu based chalcogenides, the mixing of Cu *3d* states with the chalcogenide *2p* states occur at the valence band maximum, leading to an increase in valence band dispersion. These dispersed valence bands make them the potential candidates for transparent semiconductors as they are easily affordable^3^. In some layered crystal structures, efficient mixing of Cu and chalcogenide takes place at the top of the valence bands. Such crystals also possess lower effective masses and are predicted to be useful in solar cell absorbers due to their ability to be bipolar and defect tolerant^4^.

The present study on ternary KCuX has been initiated as the layered materials^5^ exhibit a wide variety of applications in the field of contemporary optoelectronics^6–11^. The ternary alkali metal copper chalcogenides possess an enormous range of chemical formulae and can be categorized either by their crystal structures or by their electronic band structures^12^. The compounds containing copper can be classified into two general categories, where Cu is either in valence precise or in a mixed valence state. It has been established that besides copper, the chalcogen present in the ternary systems also exhibit a mixed valence state^13,14^. Valence precise state compounds are reported to be semiconductors^15^ while the mixed valence state compounds could be metals or superconductors^16^. Present ternary Zintl phases KCuX(X = Se, Te) fall into the category of valence precise compounds and these compounds were synthesized at 973 K and 1073 K respectively^5^.

Ab-initio calculations have become an effective tool to explore characteristic properties of materials and provide interpretation for experimentally observable phenomena. Density functional theory (DFT) allows having a plethora of required properties at ambient conditions as well as under the influence of external parameters. Huge efforts have been made in search of semiconductors with moderate band gaps, high carrier mobility and thermal stability for potential applications in photonic devices and nanoelectronics. When it comes to microelectronic semiconductors, carrier mobility plays a key role in the charge transport properties. So, we conceived an idea of testing the ternary intermetallic compounds for the said potential applications. To achieve the desired charge transport properties we have computed the electronic properties such as charge density and electronic band structure, optical properties such as dielectric function and absorption using DFT. Present ternary semiconductors have been found to have a direct band gap close to that of silicon (1.14 eV) and so are being explored in quest of new semiconductors with contemporary technological applications. Subsequently, this work also provides further information for the existing structural data on the physical properties of the materials.

In this work, we aim to present a theoretical insight into the structural, electronic, charge transport and optical properties of ternary KCuX with efficient TBmBJ potential by including spin-orbit interaction in the ab initio calculations. Present ternary systems have buckled configuration in the structure and are expected to handle more pressure and maintain the stability. So, the ternary crystals are also studied under the influence of a small pressure (5 GPa) to know the stability and flexibility of the materials. Most of the work in this paper is reported for the first time and hence there is a lack of experimental data for the comparison of demonstrated results. This study offers a fertile testing ground for the exploratory work on more unexplored intermetallic Zintl phases of this kind.

## Results and Discussion

**Figure 1:**
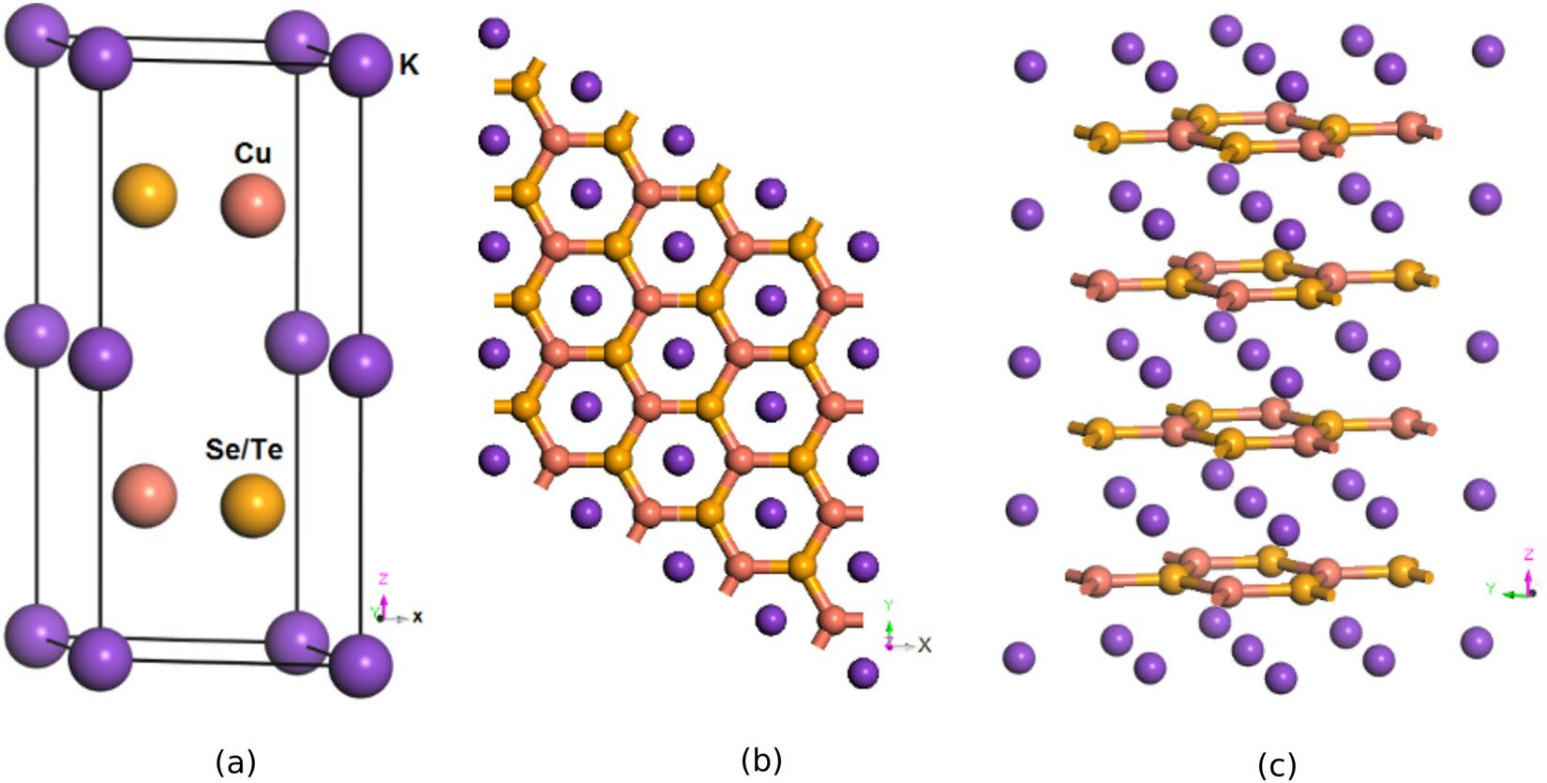
Optimized crystal structure of KCuX (**a**) A perspective view of unit cell orient towards xz plane (**b**) 2D view along xy plane showing honeycomb lattices formed by Cu and Se/Te with AB stacking and K located at the inversion center (**c**) layered structure orient towards yz plane.

The presently dealt ternary Zintl phases KCuX were synthesized by Savelsberg and Schfer in 1978^5^. This class of materials crystallize in InNi_2_ honeycomb lattice structure and exhibit hexagonal P6_3_/mmc symmetry with space group number $194{\, \mbox{-} D}_{6}^{4}$ h like our recently explored topological materials KZnX (X = P, As, Sb)^17^. The ground state crystal structures of the present non-symmorphic, centrosymmetric systems are shown in Fig. 1 where Cu and Se/Te ions form honeycomb layers with AB stacking along c axis; between each AB bilayer there exists a triangular lattice of K atoms. The K atoms are situated on top of the center of the honeycomb lattice while Cu/Se/Te atoms are positioned on top of the center of the triangular lattice. The K, Cu and Se/Te atoms are positioned at (0, 0, 0) in the A plane and at (1/3, 2/3, 1/4) and (2/3, 1/3, 3/4) positions in the B plane respectively. The calculated results on structural data could be compared with only available experimental results on crystal structure^5^.

### Ground state structural, mechanical and Electronic properties

Firstly, the experimental crystal structures were optimized under ambient conditions to achieve the stable structures. These optimized structures were further used to calculate the required properties of the respective materials. The crystallographic parameters (lattice parameters a, c, volume V) and bond lengths are displayed in Table 1. The obtained structural details at ambient conditions are in reasonable agreement with the experimental data^5^. Further calculations were carried out after confirming that the studied materials possess mechanical stability.

**Table 1:** Optimized lattice parameters, volume and percentage error w.r.t experimental data^5^ of ternary KCuX(X = Se, Te) using PBE + G06, bond lenths in (Å), band gap (E_*g*_) (GGA + TBmBJ) without and with SOC in eV.

Compound	a(Å)	c(Å)	V(Å)^3^	K-K	K-Se/Te	Se/Te-Cu	E_*g*_	E_*g*_ + SOC
KCuSe	4.21	9.79	150.67(+4.37%)	4.21	3.64	2.43	1.147	1.128
Experiment	4.18	9.54	144.35	4.18	3.39	2.41	—	—
KCuTe	4.51	10.17	179.12(+4.50%)	4.50	3.9	2.6	1.476	1.384
Experiment	4.46	9.95	171.40	4.18	3.90	2.60	—	—

The study of elastic properties of a crystal contributes to valuable information about the anisotropic character of the bonding and its structural stability. The symmetry operations in hexagonal crystals lead to five independent elastic constants C_*ij*_ (C_11_, C_12_, C_13_, C_33_, C_44_). As shown in Table 2, C_*ij*_ constants calculated using the stress-strain relationship^18^ are positive and satisfy the generalized Born’s criteria^19,20^ for mechanically stable crystals: C_*ii*_ > 0 (i = 1, 3, 4), C_11_ > C_12_, (C_11_ + C_12_)C_33_ > 2 C_13_^2^. The elastic constant C_11_ which indicates the elasticity in length, is approximately equal for both the ternary crystals, pointing towards similar melting temperature thereby confirming that KCuX can withstand temperatures as high as 1100 K. The melting temperature was calculated using the equation which involves only C_11_; *T*_*m*_ = [553 K + (5.91 K/GPa)*C*_11_] ± 300 K^21^. The bulk modulus (B), shear modulus (G), modulus of elasticity (Young’s modulus (Y) also denoted as (C_3*D*_)), mass density (*ρ*) and Debye temperature (*θ*_*D*_) are calculated using a set of elastic constants^20,22^. As seen in Table 2 the calculated elastic properties demonstrate that the systems are stable against shear owing to the smaller value of Poisson’s ratio (*σ*). As *σ* is less than 0.33 (Table 2) the materials are predicted to have brittle nature as expected in a Zintl phase.

**Table 2:** Elastic constants, bulk (B), shear (G) and youngs modulii (Y) (GPa), Poissons ratio (*σ*), Debye temperature (*θ*_*D*_) (K), melting temperature (T_*m*_) (K) and mass density (*ρ*) (g/cc) of KCuX(X = Se, Te).

Compound	C_11_	C_33_	C_44_	C_12_	C_13_	B	G	Y	*σ*	*θ*_*D*_	T_*m*_	*ρ*
KCuSe	109.2	55.27	27.22	44.31	24.82	44.02	28.16	71.82	0.249	302.55	1198.4	4.00
KCuTe	105.67	44.21	23.63	26.75	20.6	36.16	26.67	68.57	0.213	273.08	1178.5	4.26

**Figure 2:**
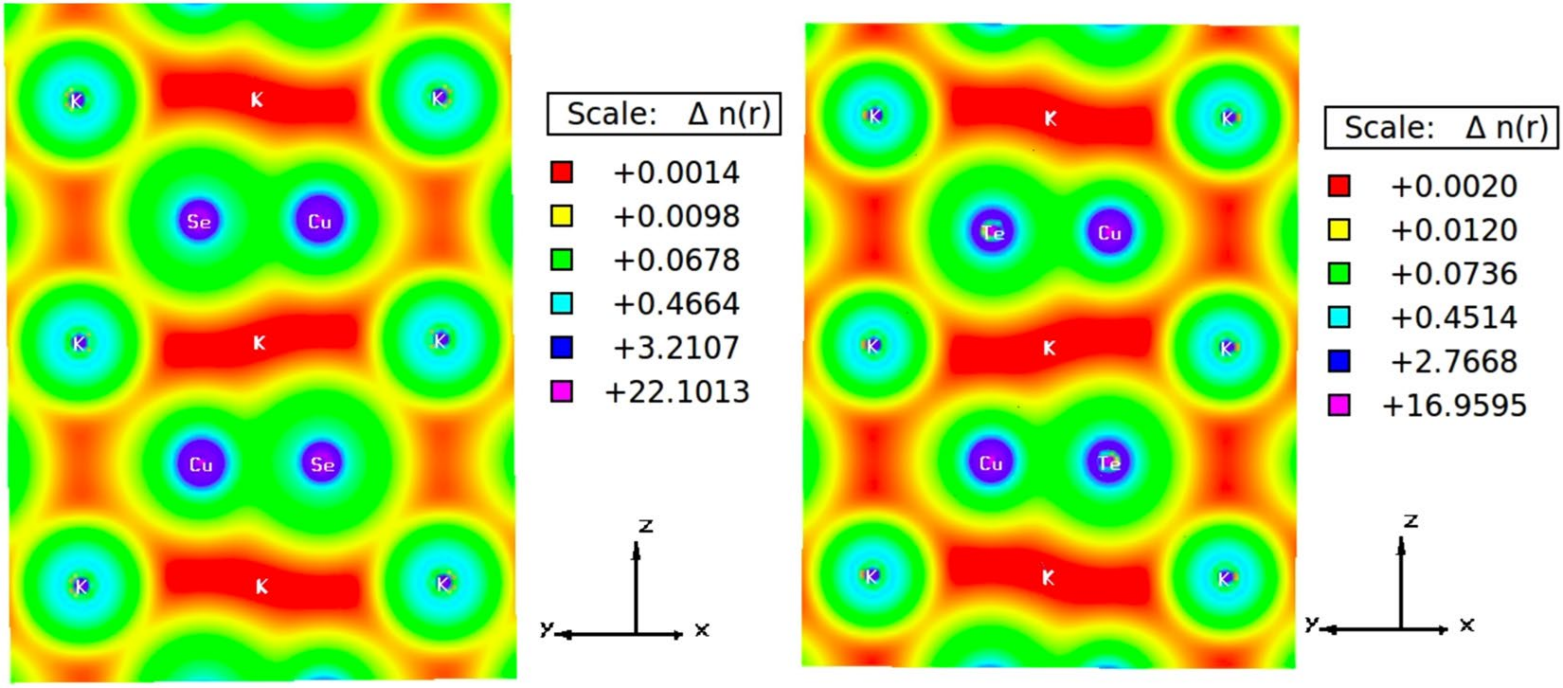
Computed charge density indicating Cu-Se/Te covalent bonding for (left) KCuSe and (right) KCuTe at ambient conditions.

The chemical bonding nature amid two atoms can be predicted from the electronegative difference. According to Pauling scale^23^, the electronegativity values for K (0.82), Cu (1.9) and Se/Te (2.55/2.1) give the electronegative difference as 1.73/1.28, 1.08 and 0.65/0.2 for K-Se/Te, K-Cu, Cu-Se/Te bonds respectively. The electronegative difference indicates that the bonds associated with K and Cu are covalent while those associated with K and Se are covalent and slightly ionic. This type of peculiar bonding is expected in these systems as most of the intermetallic Zintl phases possess mixed bonding. Electronic charge density plots explain the accurate bonding nature of a material, where charge transfer between ions indicates an ionic nature and sharing of charges between ions shows a covalent nature in the crystal. The charge density difference plots of these compounds are shown in Fig. 2 along (1 1 1) crystallographic plane. It can be seen that a denser charge cloud is distributed from Cu to Se/Te indicating covalent bonding similar to what electronegative difference suggested, thereby making KCuX viable in future solid state device applications.

**Figure 3:**
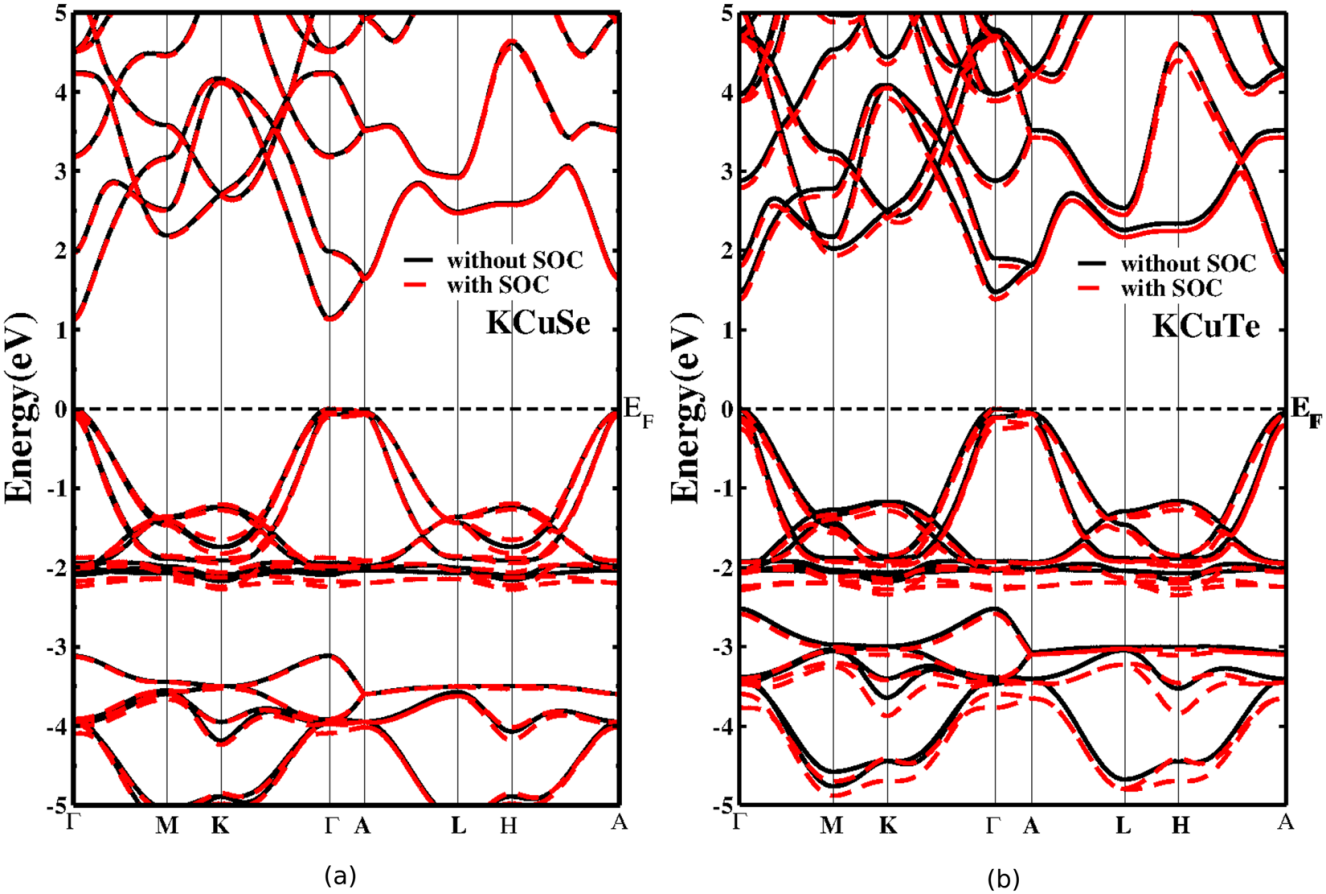
Calculated electronic band structure of optimized crystal at ambient conditions using TBmBJ potential without and with spin-orbit coupling for (**a**) KCuSe and (**b**) KCuTe with the Fermi level fixed to zero.

As the conversion efficiency required in a photovoltaic material is closely related to its electronic band gap, calculating reliable band gaps of present ternary phases has become more essential. Standard semi-local GGA underestimates the band gaps of semiconductors as quasi-particle excitations are not considered in DFT; the exchange and correlation effects of self-consistency are therefore treated by TBmBJ potential^24^ by incorporating the relativistic spin-orbit interaction in order to find reliable band gaps and band shapes. The calculated electronic band gap (Eg) values for KCuSe (1.128) and KCuTe (1.384) are ideal for photovoltaic applications^25^. Figure 3 presents the calculated band structures for KCuX, which clearly depicts that the crystals are direct band gap semiconductors as both the conduction band minimum (CBM) and the valence band maximum (VBM) are located at the gamma point; thus a vertical transition is allowed for optical absorption. The Fermi level is arbitrarily shifted to zero and is shown by the horizontal dashed line. We note that the highest occupied state in the band structures of KCuX at the gamma point is doubly degenerate as their crystal structures belong to hexagonal symmetry in the dihedral point group. The lower conduction bands are mainly composed of *s* and *p* states of K, Cu, Se/Te, while the highest valence band mainly consists of Cu-*p*, Se/Te-*p*, *d* states. The group I cation K does not influence the makeup of the VBM which is solely dominated by Cu and X states. The results on electronic band structure elucidate that spin-orbit coupling (SOC) leads to the non-local band splitting around 2 and 4 eV in the valence band. It can be noticed that flat valence band edge is less hybridized than steeper conduction band edge in both the materials. In order to illuminate the bonding situation and the effect of atomic relaxation on electronic band structures, we have calculated the partial density of states (pDOS) without and with SOC effect (Fig. 1 in Supplementary Data). Consistent with the band structure, pDOS also reveal that in the region near Fermi level, the CBM is predominantly occupied by *p*, *s* states and some contributions from K-*p*, Cu-*p*, *d* and Se/Te-*p*, *d* orbitals can be seen respectively. The VBM, on the other hand, is predominantly filled by Cu, Se/Te-*p* states and some contributions from Cu, Se/Te-*d* states are noticed. In addition, it is observed, as expected, that K shows the minimal contribution to the CBM and VBM. The unoccupied bands near the band gap edge indicate a strong hybridization of *s* orbitals. The highest probability of transition in KCuX(X = Se, Te) is from Se-*p* states to Cu-*s* states and Cu-*p* states to Te-*s* states respectively.

### Effective Mass, Carrier Mobility and Optical Properties

Effective mass is an important material parameter which can describe most of the carrier transport properties in semiconductors^26,27^. The precise knowledge of effective mass becomes more important when it comes to the technology of optoelectronic devices. The value of electron effective mass at CBM and hole effective mass at the VBM was obtained from the curvature of respective bands at the gamma point^28^. For holes, one should note that there are two valence bands near the gamma point as VBM is a doubly degenerate state. So, two kinds of holes occur, which are termed as light and heavy holes respectively. The larger the band curvature smaller the effective mass and this condition proves that the valence bands are flatter than conduction bands in KCuX. As shown in Table 3, the electron effective mass is similar but the light hole and heavy hole effective masses are greater for KCuSe. When compared with the conventional semiconductors^27^ KCuX have significantly small effective mass value, making them promising materials for electronic devices. When photoelectrons are produced in a solar cell, recombination of electrons and holes decreases the solar conversion efficiency. To overcome this issue of electron-hole recombination, large carrier mobility is needed^29^. So, we investigated the charge carrier transport properties of KCuX with a deformation potential approximation^30,31^. The carrier mobility (*μ*_3*D*_) gives an intuitive description in the carrier transport of a 3D solid and can be written as:

$${\mu }_{3D}={2}^{3/2}{\pi }^{1/2}{{\rm{C}}}_{3D}{\hslash }_{e}/3{{{\rm{E}}}_{1}}^{2}{{\rm{m}}}^{\ast }{}^{5/2}{({{\rm{K}}}_{B}T)}^{3/2}$$

where C_3*D*_ denotes the modulus of elasticity, E_1_ refers to the deformation potential obtained by varying the lattice constants and studying the change of band energy under the lattice compression and strain. The calculated deformation potential (E_1_) and carrier mobility is presented in Table 3. Effective mass strongly influences the carrier mobility; the smaller the effective mass, larger the carrier mobility in the case of KCuX, *μ*_3*D*_ is as high as 10^5^ cm^2^/Vs. This range of carrier mobility is very high compared to the semiconductors used in photovoltaic materials^32,33^. Due to lack of literature on these materials, we have compared the calculated mobility values with graphene which is relevant to present materials in terms of structure and technological applications. The calculated mobility values of KCuX (Table 3) are higher than the reported experimental (1.2 × 10^5^ *cm*^2^/Vs) and theoretical (2 × 10^5^ *cm*^2^/Vs) values of graphene as well as narrow band gap semiconductors h-Si_6_ (in order of 10^3^ *cm*^2^/Vs) and hexagonal boron phosphide (in order of 10^3^–10^4^ *cm*^2^/Vs) at room temperature^27,34–37^. The calculated low effective mass leads to high mobility in KCuX and effective mass results also indicate that KCuTe has more dispersive bands than KCuSe which makes the latter a better-suited material for photovoltaic applications. This prediction is also supported by the carrier mobility results as electrons in KCuTe have the highest mobility indicating that it is high electron conductive material at ambient conditions.

**Table 3:** Effective mass in m_0_, deformation potential (E_1_) (eV) and carrier mobility (*μ*_3*D*_) (10^5^ *cm*^2^/Vs) for KCuX(X = Se, Te).

Compound	Carrier Type	m^*^	E_1_	*μ*_3 *D*_
KCuSe	electron	0.229	0.253	28.49
	light hole	−0.432	0.179	11.64
	heavy hole	−0.501	0.175	8.41
KCuTe	electron	0.232	0.21	38.13
	light hole	−0.253	0.17	25.29
	heavy hole	−0.332	0.173	22.95

**Figure 4:**
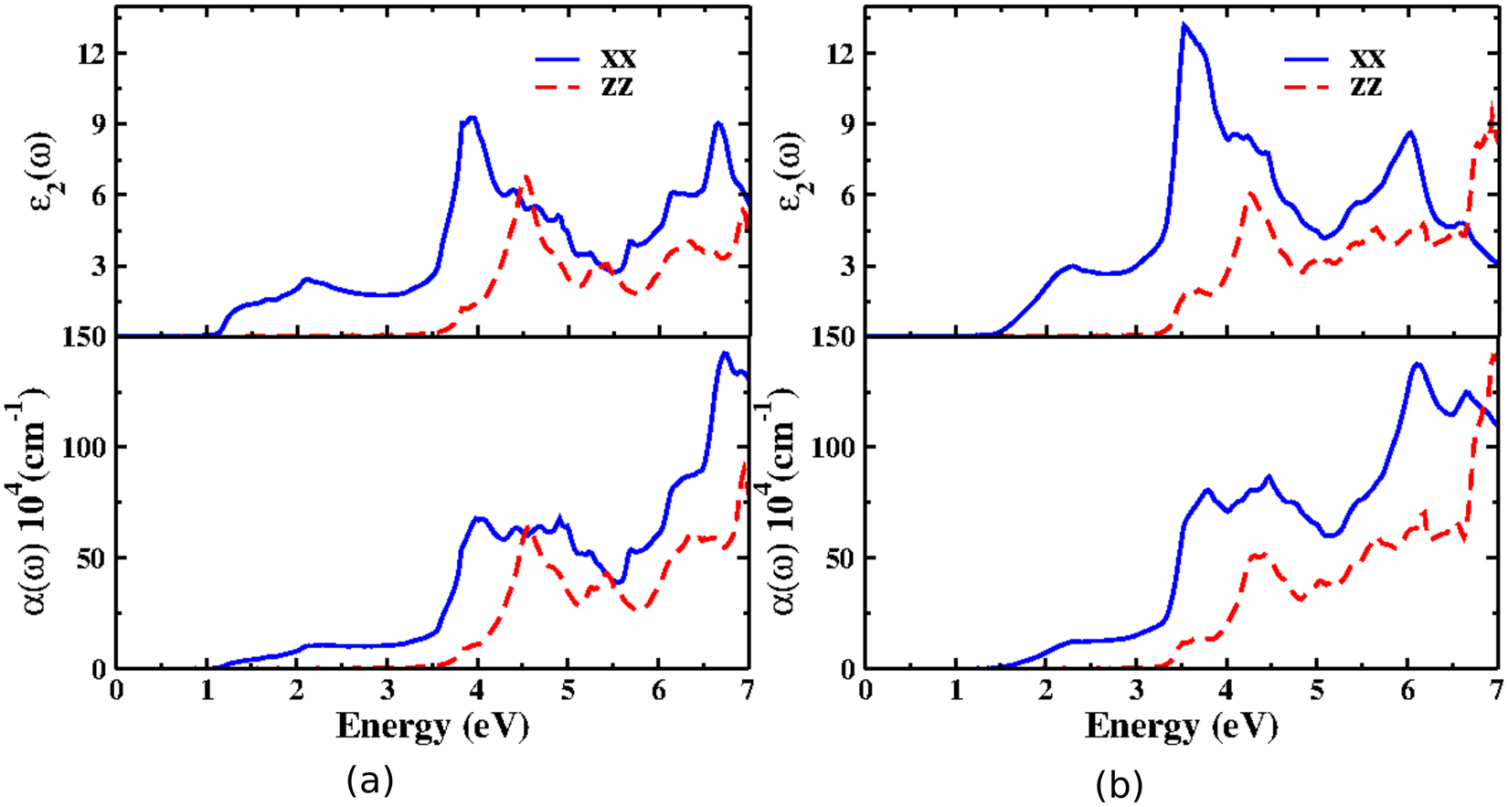
Optical properties: Profiles of imaginary part of dielectric function and optical absorption coefficient for (**a)** KCuSe and (**b**) KCuTe along xx and zz with SOC.

Further, we present the calculated optical properties of KCuX by employing TBmBJ potential with SOC effect. The optical anisotropy along x and z directions indicates the layered structure of the material. It is examined through calculated optical constants like dielectric function and optical absorption along different crystallographic axes for the photon energies up to 7 eV. The complex dielectric function describes the linear response of the system towards electromagnetic radiation. As the present ternary crystal systems crystallize in the hexagonal symmetry, the non zero components of the dielectric tensors are allowed along [100] and [001] crystallographic directions which are represented by xx and zz directions respectively as displayed in Fig. 4.

The calculated dielectric functions exhibit three prominent peaks as the function of photon energy till 7 eV. The peaks in the imaginary part of dielectric function arise mainly due to electric-dipole transitions between valence and conduction bands. The first peak around 2 eV, second around 4 eV and the third around 6.8 eV along x-direction and the three peaks along z-direction which appear at slightly higher energies are all attributed to interband transitions between Se-3d and Cu-*4s*, 3*s* states of Cu and *4p* states of Se respectively in KCuSe. At the same time, the three major peaks in KCuTe occur at 2.3 eV, 3.5 and 6 eV owing to interband transitions between Se-*3d* and Cu-*4s*, Se-*3p* and Cu-*3s* states respectively. The optical absorption spectrum was calculated in the independent particle approximation by neglecting excitonic effects. It is noteworthy that the optical absorption shows significant anisotropy where the light is absorbed more strongly along a and b axis polarizations except at 4.5 eV and 7 eV along c-direction. Regardless of polarization, the absorption spectra are relatively weak from the onset at the direct band gap emphasizing that the materials possess good carrier transport to realize their probable application in solar cell absorbers. Besides, the absorption spectra show that KCuX are sensitive to UV-A region^38^ indicating that the materials could be useful in photovoltaic and photodetector applications. Interaction of UV radiation with matter causes possible low and high energy electronic transitions. In present crystals, the electronic transition requires an absorption of a photon with a wavelength which falls in UV-A range. Therefore, the origin of the peaks in absorption spectra is due to low energy *π* → π^*^ and high energy *σ* → σ^*^ interband transitions. This work predicts that the ternary KCuX semiconductors could be suitable for photovoltaic solar cell and optoelectronic devices due to large absorption coefficients and medium band gaps suitable for the said applications^39,40^. However, in order to further explore the materials based on these applications, a clear understanding of the band gap and charge transport properties under external conditions such as pressure is necessary.

### Effect of Pressure on Structural, Electronic and Charge Transport Properties

We have demonstrated the influence of applied hydrostatic pressure (0–5 GPa) on the crystal structure of KCuX as the materials possess the buckled honeycomb lattice configuration and can withstand trivial pressure^17,41^ without having any structural phase transitions. As revealed in Fig. 2 of Supplementary Data the normalized lattice parameters and volume decrease monotonically without any anomaly. This confirms that the materials do not exhibit any structural phase transition under pressure up to 5 GPa. Further, it is observed that the reduction in ‘c’ with the increase in pressure has a major contribution in lowering the volume of the crystals. For more precise observation about change in volume a calculated P-V data is plotted which indicates a reduction in the volume (Fig. 3 in Supplementary Data) owing to decrease in Cu-Cu and X-X bond distances as shown in Fig. 4 of Supplementary Data. Intramolecular bond lengths K-K, K-Se/Te and Se/Te-Cu also decrease monotonically with an increase in pressure indicating that the bonds become more stiffer under pressure. The bond distance between K-Se/Te is the most compressible and is prominently responsible for the increase in electronic band gaps of the materials.

**Figure 5:**
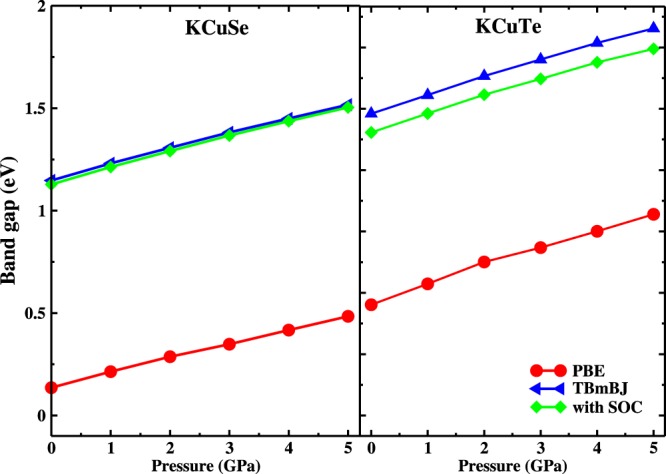
Effect of pressure (0–5 GPa) on band gaps using PBE, TBmBJ potentials and by incorporating SOC. The band gaps remain direct (at Γ) under the influence of pressure.

**Figure 6:**
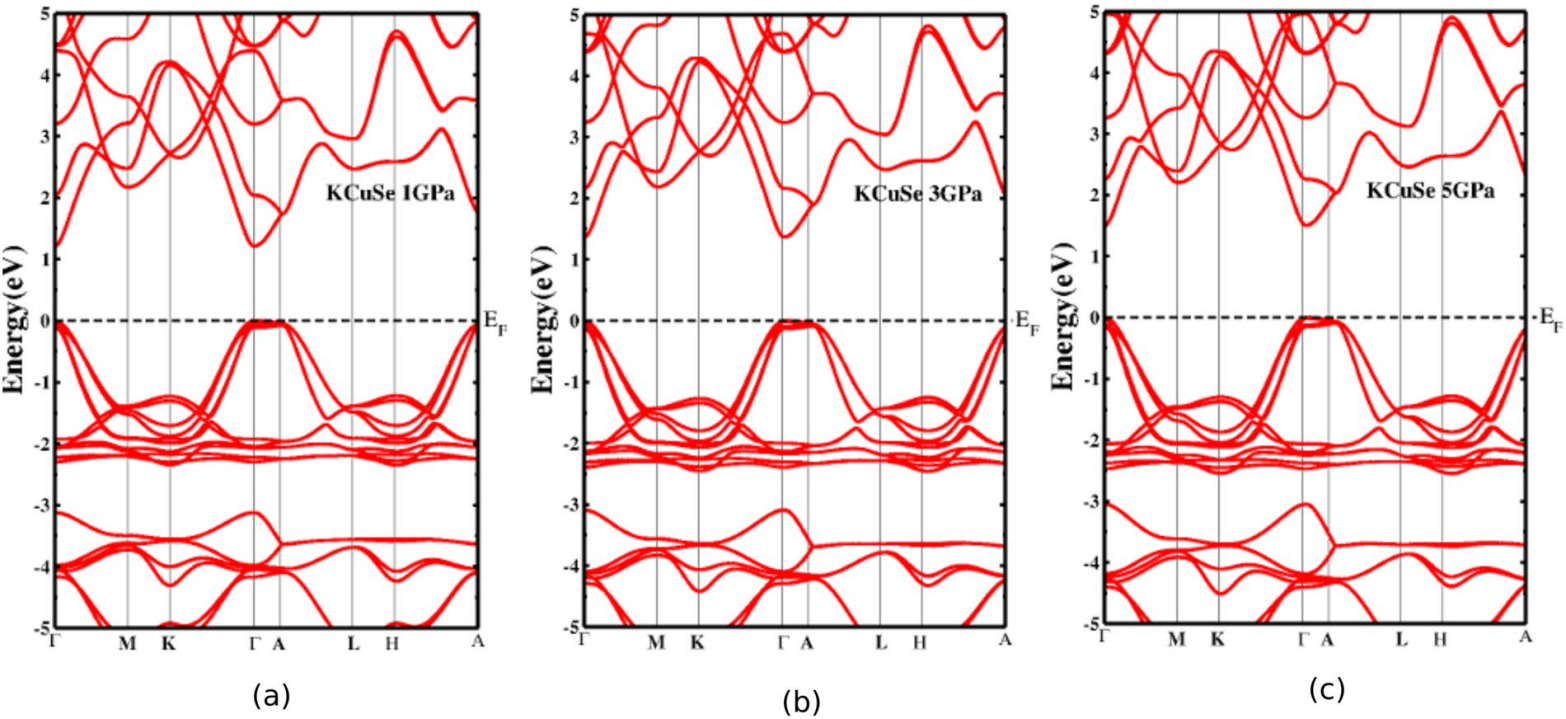
Effect of pressure on band structures of KCuSe at (**a**) 1 GPa (**b**) 3 GPa and (**c**) 5 GPa by using TBmBJ potential and incorporating SOC.

**Figure 7:**
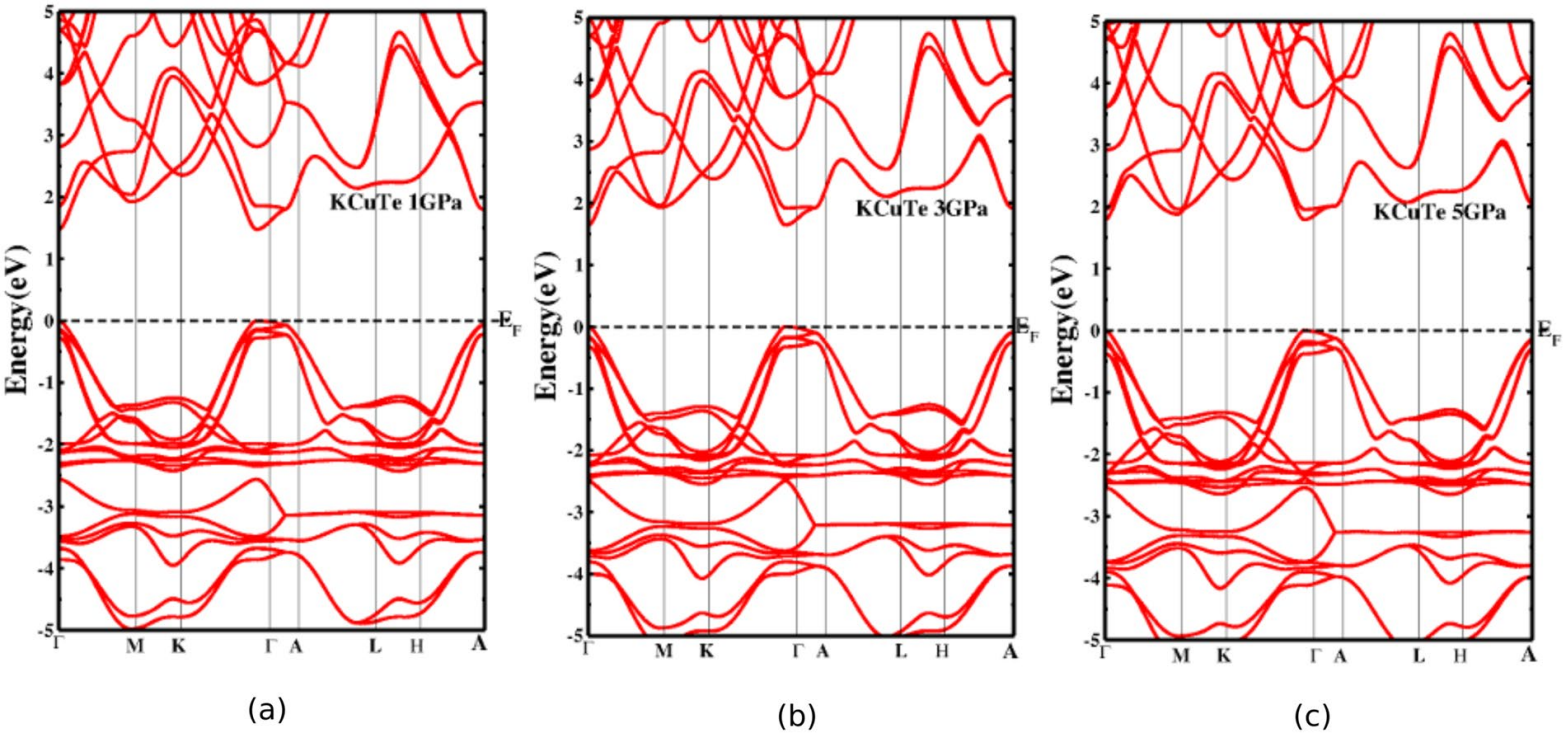
Effect of pressure on band structures of KCuTe at (**a**) 1 GPa (**b**) 3 GPa and (**c**) 5 GPa by using TBmBJ potential and incorporating SOC.

The band gaps are calculated under pressure consistently with GGA, TBmBJ potentials with and without the inclusion of SOC as displayed in Fig. 5. Under pressure, the interlayer distance between the atomic planes decrease, the inter and intralayer interaction may alter the electronic properties of the materials. It is inferred that in present ternary KCuX, the band gaps are sensitive to pressure and increase linearly with an increase in pressure like in other semiconductors^42–44^. This increase in band gap as a function of pressure promotes the structural stability of the present crystal systems^45^. The increase in band gaps also suggests a shift in absorption curve in the absorption spectra. Further, the band gaps decrease slightly when SOC is turned on with TBmBJ potential. The band gap values calculated using TBmBJ are more than twice in order of magnitude with GGA for both crystal systems. The SOC has a minimal effect which apparently increases with the X radii. The heavier the X atom, the more the effect of SOC. The difference in band gap without and with the inclusion of SOC varies from 0.02 to 0.09 for KCuSe and KCuTe respectively. This difference validates the minimal effect of SOC on optoelectronic properties of KCuX. The impact of pressure on electronic band behavior was probed as is displayed in Figs 6 and 7. It is interesting to note that the conduction and valence bands are pushed away from Fermi level under pressure hence, the band gap increases with an increase in pressure.

**Figure 8:**
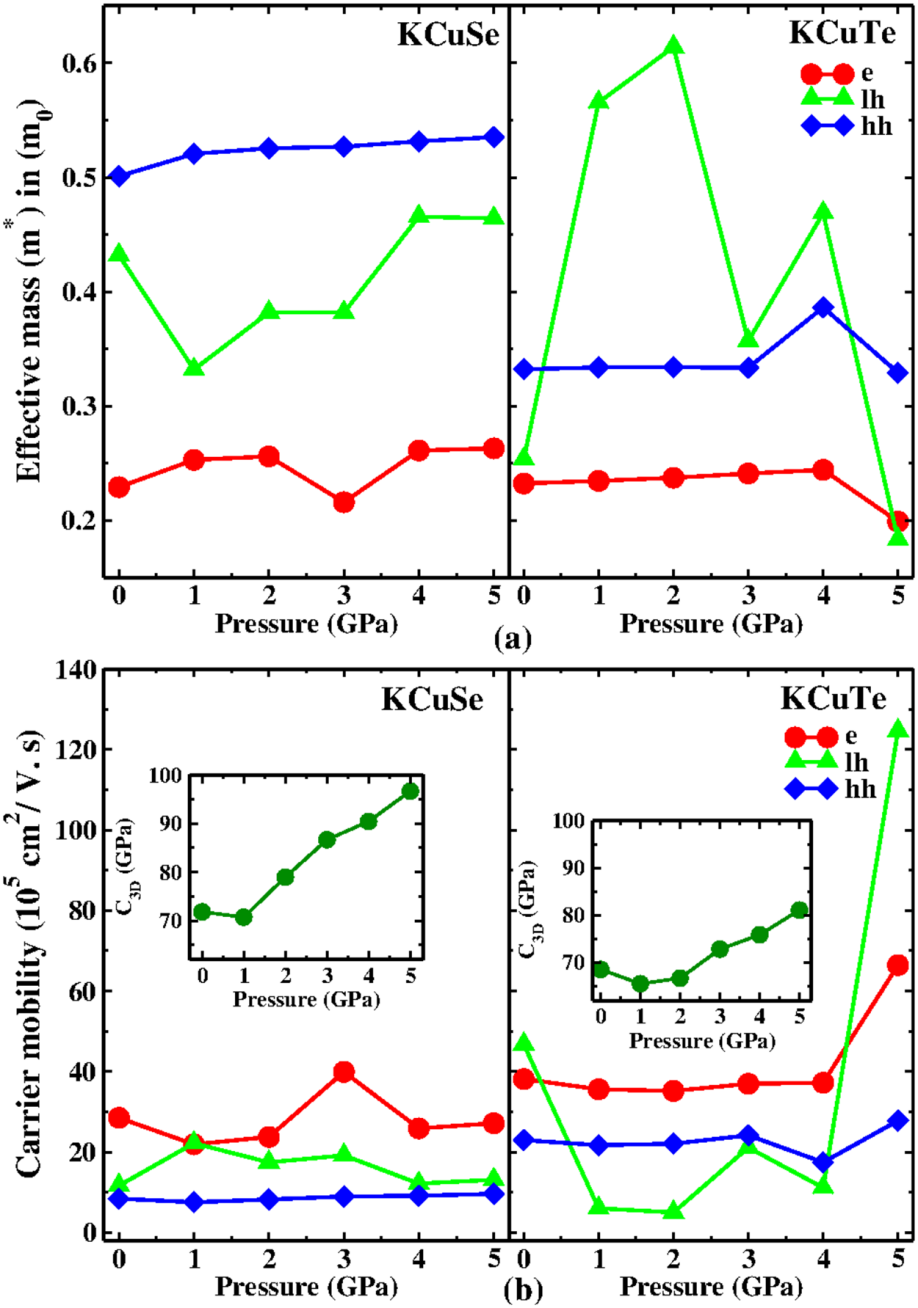
Effect of pressure (0–5 GPa) on (**a**) Effective mass (m^*^ in m_0_) and (**b**) Carrier mobility and elastic modulus (inset) for KCuX(X = Se, Te).

The search for photovoltaic and solar cell absorption applicability of the present materials is extended by determining the electron and hole effective mass under pressure. Effective mass values in Fig. 8(a) emphasize an interesting relation here: the effective mass for KCuSe (which has lower band gap) is higher, confirming that the top of the valence band and bottom of the conduction band are clearly “flatter” than those of KCuTe at 5 GPa, since the effective mass is proportional to the inverse of band curvature. The electron effective mass for KCuSe exhibits fluctuating behaviour under the influence of pressure where as for KCuTe it shows a slight linear increase till 4 GPa and drops at 5 GPa with a difference in magnitude of about 0.045 m_0_. This further confirms that under varying pressures KCuTe may possess better transport properties. The carriers in KCuTe exhibit a low band mass, indicating a pronounced parabolic nature of bands at gamma except for the light hole band mass which oscillates from the highest to the lowest values (0.614 to 0.183 m_0_). The light hole valence band shows anisotropic effective mass which may be due to the layered structure of the crystal systems, nevertheless it has a minimal role in the carrier transport properties.

An informative insight into the transport properties can be gained by understanding the effects of pressure on semiconductors. And, mobility is a key quantity in electron transport as it defines the motion of a charge carrier upon an applied bias. Furthermore, motivated by the technological need for high mobility and keeping in mind that the effective mass can very well describe the mobility, we have calculated the charge carrier mobility under pressure as an external influence using the deformation potential approximation^30,31^ for the present ternary semiconductors. The modulus of elasticity has a major role to play in determining the receptivity of the material towards physical changes. The obtained modulus of elasticity is sensitive to pressure and increases with pressure except at 1 GPa as displayed in the inset of Fig. 8(b). In addition, the melting temperature is found to increase with pressure which is 1414 K and 1302 K (at 5 GPa) for KCuX respectively, pointing towards the reinforcing thermal stability under the influence of pressure. As seen in Fig. 8(b), the obtained carrier mobility improves under pressure and specific higher values are achieved by electron and hole mobilities of KCuTe making it a better electron conductive material at high pressure as well. Also, Fig. 8 illustrates that the low effective mass leads to high mobility in these crystal systems as is reported earlier for other semiconductors^6,46,47^.

The studies on the effect of pressure on the said properties indicate that pressure is effective in tuning the electronic band structure and carrier mobility of KCuX. It is expected that pressure can have a significant impact on a wide range of physical properties crucial for their potential applications.

## Conclusion

The ground-state structural, mechanical and optoelectronic properties of the unexplored centrosymmetric KCuX(X = Se, Te) are systematically studied using first principles calculations for the first time. It is emphasized from ab initio calculations that the ternary Zintl phases KCuX are direct band gap semiconductors and UV-A light absorbers. It follows from the flat electronic bands along the Γ-A direction and strong optical anisotropy that the materials are quasi two-dimensional. Besides, we reveal that the materials exhibit good light absorption, possess low effective mass and high intrinsic mobility. Pressure effectively tunes the electronic band structure which has a substantial impact on electronic and transport properties. This work presents the state-of-the-art theoretical calculations and predominantly highlights that the ternary KCuX(X = Se, Te) systems own all the desirable electronic, optical and charge carrier transport properties which most probably make them not only feasible materials as solar cell absorbers but also useful in photovoltaics, photodetectors and other contemporary optoelectronic applications. The insights and parameters reported here shall be useful in the interpretation of experiments on these materials or their prototypes and would also direct researchers towards device modeling.

## Methods

Structural optimizations were carried out using the pseudopotential method implemented in the plane wave self-consistent field (Pwscf) program^48^. Generalized gradient approximation (GGA) in the Perdew-Burke-Ernzerhof (PBE) parametrization using norm-conserving pseudo-potential approach was considered to treat electron-electron interactions with the plane wave energy cut off of 350 Ry. The calculated lattice parameters and bond distances (Table 1) are in fair agreement with the reported experimental data^5^. The band structure calculations were performed using the FLAPW method, as implemented in WIEN2K^24^. The plane wave cut-off value in the interstitial region for R_mt_ K_max_ was set as 7.0. The separation energy between the valence and core states was chosen to be 6.0 Ry. Integrations in the Brillouin zone were performed using k-points generated with 13 × 13 × 5 mesh points for the computation of electronic properties. Optical properties were calculated based on electric dipole transitions in the independent particle approximation with a denser k-mesh of 29 × 29 × 11 as implemented in the WIEN2k code^49^.

## Electronic supplementary material

Supplementary Information

## Data Availability

All data generated or analysed during this study are included in this published article(and its Supplementary Information file).
