# Supplementary material for: Exploring Exemplary Optoelectronic and Charge Transport Properties of KCuX(X=Se,Te)
Source: Sci Rep. 2018 Aug 30;8:13071. doi: 10.1038/s41598-018-31300-0 (PMC6117315; doi:10.1038/s41598-018-31300-0)
Supplement: Supplementary file 1 — Supplementary Information [file 41598_2018_31300_MOESM1_ESM.pdf]

Exploring Exemplary Optoelectronic and Charge Transport Properties of KCuX(X=Se,Te)

Atahar Parveen [1,*] **and G. Vaitheeswaran**[1]

[1]Advanced Centre of Research in High Energy Materials (ACRHEM), University of Hyderabad, Prof. C. R. Rao Road, Gachibowli, Telangana, Hyderabad-500046, India.
[*]corresponding.ataharparveen@gmail.com

ABSTRACT

We report the electronic structure, optical and charge transport properties of the unexplored ternary Zintl phases KCuX(X=Se,Te) from the first principles calculations employing the full-potential linearized augmented plane-wave (FLAPW) method with the Tran Blaha modified Becke-Johnson (TBmBJ) potential. It is demonstrated that the materials are direct band gap (1.13, 1.4 eV) semiconductors with covalent bonding between Cu and (Se/Te). The calculated low effective mass and high carrier mobility (over $10^5 cm^2/$V.s) accentuate that KCuX have good carrier transport and the materials may have possible applications in solar cell absorbers and nanoelectronic devices. Absorption spectra indicate that the ternary crystals are UV-A light absorbers and could be useful in photovoltaic and photodetector applications. A study on the effect of pressure (till 5 GPa) is carried out in order to further explore the materials for their electronic band gaps and charge transport properties as they are proposed to be useful in future contemporary electronic devices. It is observed that pressure enhances the intrinsic carrier mobility and thermal stability of KCuX, indicating that the materials can withstand robust external conditions.

.
.
.
.

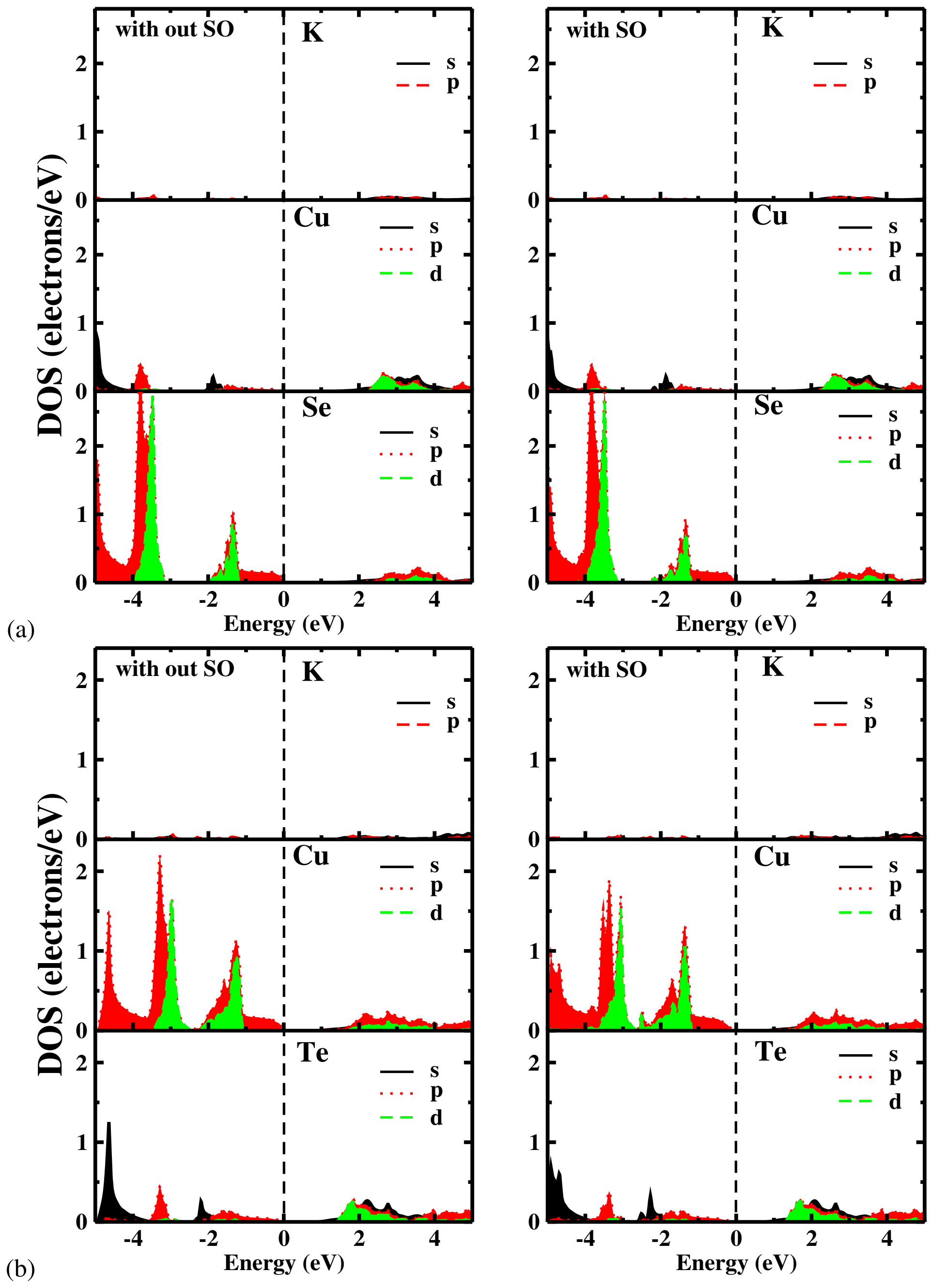

Figure 1. (Color online) Calculated partial Density of states without and with SOC for (a) KCuSe and (b) KCuTe at optimized ground state.

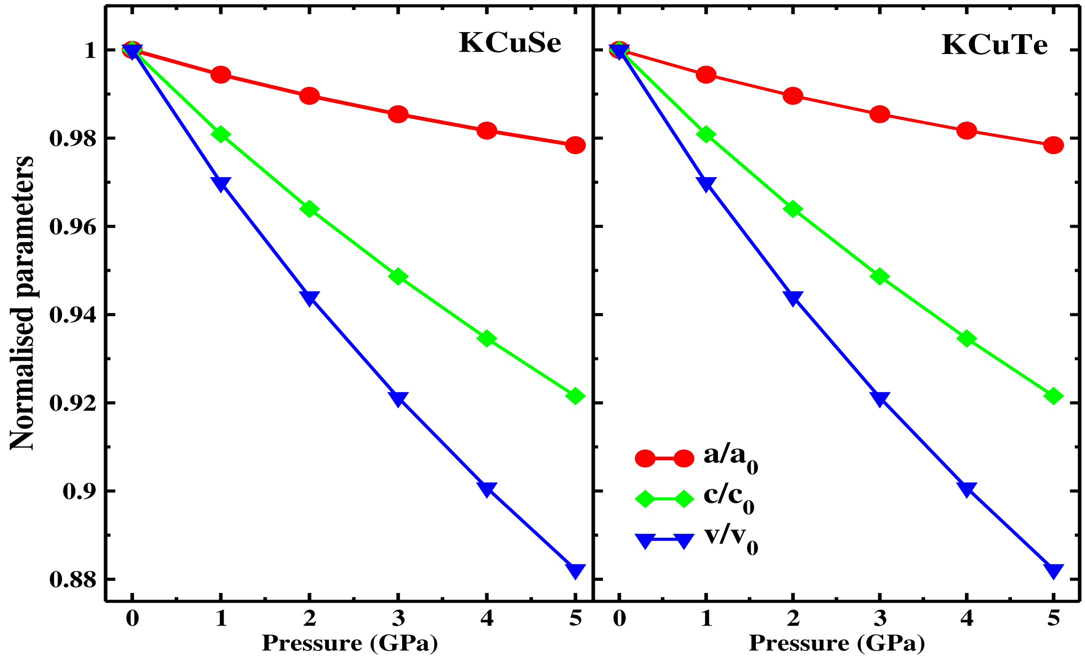

Figure 2. (Color online) Effect of pressure (0-5 GPa) on normalized parameters lattice parameters for KCuX(X=Se,Te)

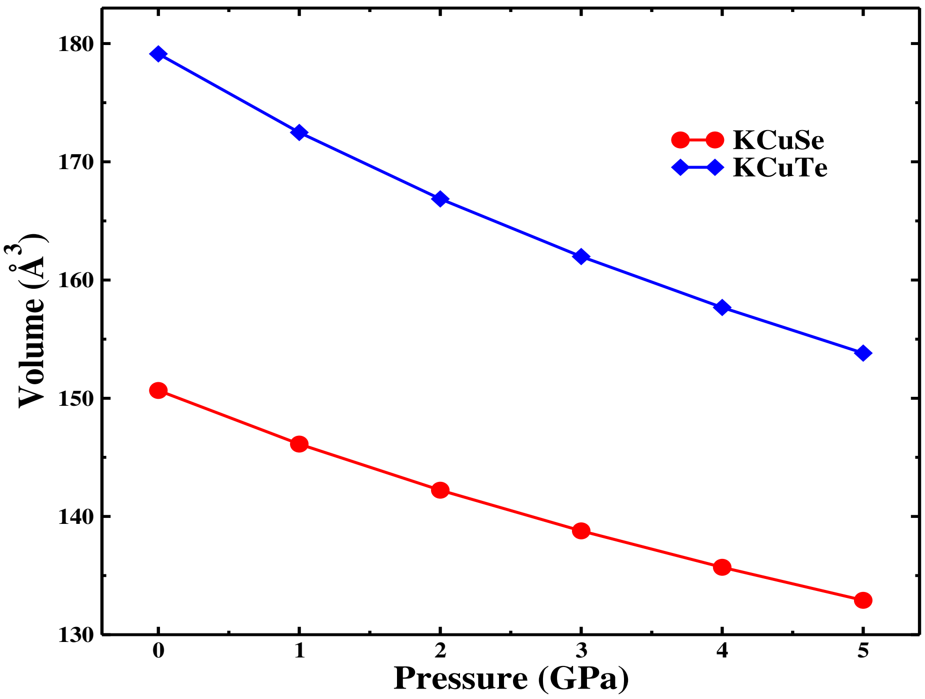

Figure 3. (color online) Effect of pressure (0-5 GPa) on volume for KCuX(X=Se,Te)

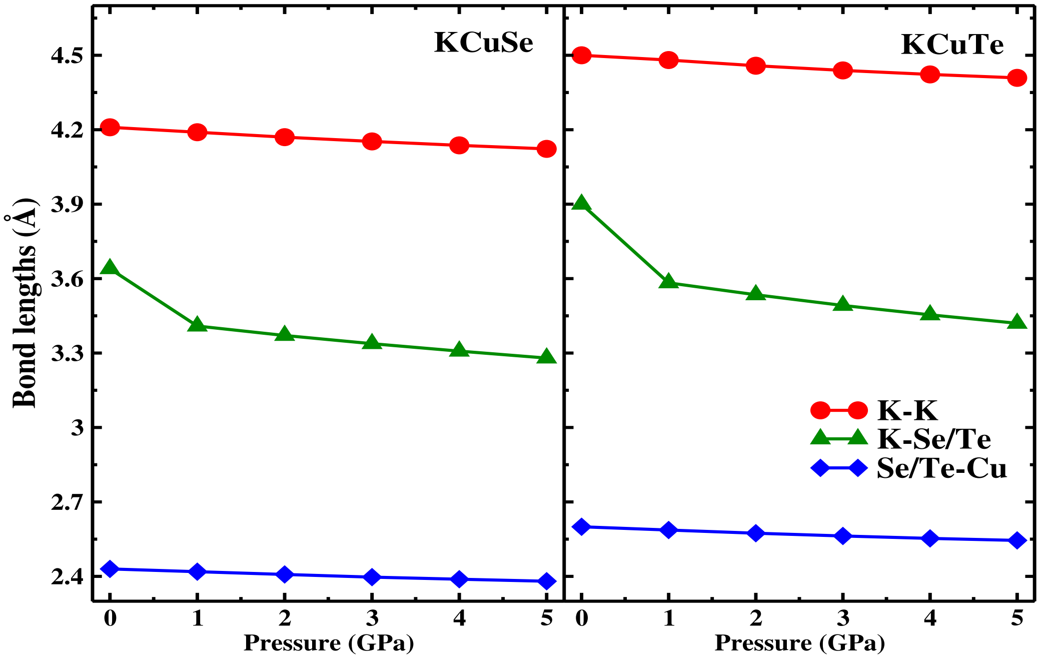

Figure 4. (Color online)Effect of pressure (0-5 GPa) on bond lenths in KCuX(X=Se,Te)
